# Supplementary material for: Acetylation-dependent coupling between G6PD activity and apoptotic signaling
Source: Nat Commun. 2023 Oct 5;14:6208. doi: 10.1038/s41467-023-41895-2 (PMC10556143; doi:10.1038/s41467-023-41895-2)
Supplement: Supplementary file 4 — Description of Additional Supplementary Files [file 41467_2023_41895_MOESM4_ESM.docx]

File Name: Supplementary Movie 1

Description: Simplified animation describing the structural differences between WT G6PD and K89- or K403-acetylated G6PD.
